# Research on Seismic and Self-Centering Performance of SMAF-ECC Prefabricated Self-Centering Frame Joints Based on Finite Element Simulation

**DOI:** 10.3390/ma19010110

**Published:** 2025-12-29

**Authors:** Yan Cao, Qing Wu, Zhao Yang

**Affiliations:** 1 College of Creative Design, Wuchang University of Technology, Wuhan 430223, China; andycaoyan@163.com; 2 School of Urban Construction, Wuhan University of Science and Technology, Wuhan 430065, China; qinng28@163.com

**Keywords:** SMAF-ECC composite materials, prefabricated self-centering frame joints, self-centering performance, seismic performance, finite element analysis

## Abstract

To address poor seismic performance, large residual displacement, and insufficient self-centering capacity of prefabricated frame joints in building industrialization, this study proposes a novel self-centering prefabricated frame joint reinforced with shape memory alloy fiber (SMAF)–engineered cementitious composite (ECC) composites (SMAF-ECC). A validated finite element model of the proposed joint was established using ABAQUS, with comparative analyses conducted against conventional reinforced concrete (RC) and ECC-strengthened (RC-E) joint models to explore the effect of SMAF volume content on seismic performance. Results show that replacing the joint core zone concrete with SMAF-ECC significantly enhances the joint’s seismic and self-centering capabilities, reducing residual displacement and optimizing hysteretic behavior. SMAF volume content is a key factor affecting performance, with an optimal value identified and excessive content leading to fiber agglomeration and degraded self-centering ability. This study provides a feasible solution to improve the seismic resilience of prefabricated frame joints, laying a foundation for the application of SMAF-ECC in prefabricated structures.

## 1. Introduction

Building industrialization drives the vigorous development of prefabricated buildings. Compared with traditional cast-in-place concrete frames, prefabricated concrete frame structures have poorer integrity and seismic performance, and joint failure is a key factor causing structural damage or collapse in earthquakes [1]. Thus, improving the seismic performance and toughness of joints is critical for ensuring structural safety. Although extensive research has been conducted on the seismic performance of prefabricated joints [2,3,4,5,6], traditional concrete’s inherent defects (poor tensile property, brittleness) lead to unresolved issues such as insufficient durability, inadequate deformation, energy dissipation capacity, and significant residual deformation in prefabricated joints [7]. Therefore, substituting traditional concrete with high-performance composites is a feasible solution to address these problems.

Engineered Cementitious Composite (ECC) is a high-ductility short-fiber-reinforced cementitious material. Based on fracture mechanics and micromechanics, its microstructure is purposefully designed and regulated. It exhibits ultra-high toughness, excellent shear ductility, superior energy dissipation capacity, and high damage tolerance [8], thus being widely used to improve the seismic performance of structures. Research has demonstrated that incorporating ECC into the core area of frame joints and the plastic hinge area of beams can reduce the dosage of stirrups and enhance the shear capacity of the joint core area. Compared with traditional concrete frame joints, those with ECC integrated into their core area not only possess higher bearing capacity and energy dissipation capacity, but also exhibit better deformation capacity and damage resistance [9,10,11]. However, these joints still suffer significant residual deformation after earthquakes, which hinders post-earthquake repair and functional restoration [12]—this constitutes a critical research gap that needs to be addressed.

**Figure 1:**
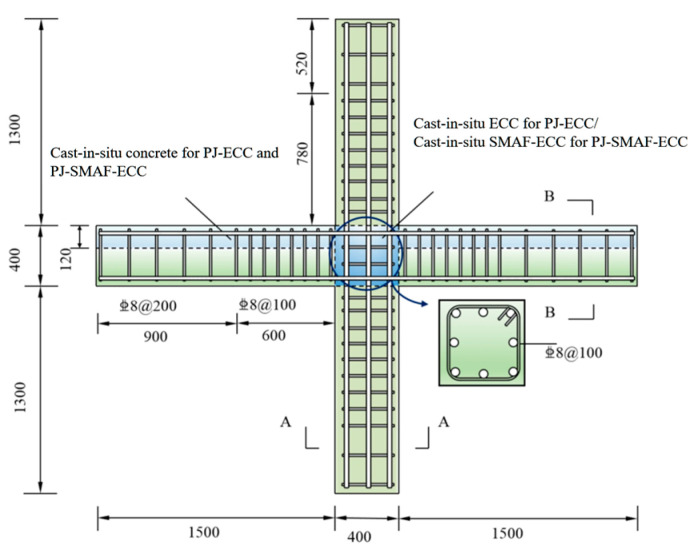
Details of SMAF-ECC joint specimens [19], unit: mm.

Shape Memory Alloy (SMA) is a functional material with unique shape memory effect and superelasticity. Among them, superelasticity is stress-induced and manifests during the loading-unloading cycle. Its superelastic flag-shaped hysteretic energy dissipation and deformation recovery characteristics enable seismic structures to possess both energy dissipation and self-centering mechanisms [13,14,15]. However, existing SMA-based solutions predominantly use continuous SMA materials (e.g., strands or rods), their high cost and requirement for specialized anchoring devices severely limit engineering applications this is a key insufficiency of current SMA-based technologies. Shape Memory Alloy Fiber (SMAF), by contrast, has fewer defects, requires no cutting, simplifies manufacturing, and has lower costs, making it more suitable as an ECC reinforcement. Randomly distributed SMAF in ECC forms SMAF-ECC composites with crack closure, deformation recovery, and excellent energy dissipation capacity [16,17,18]. Applying SMAF-ECC to prefabricated joint core areas and beam-column plastic hinge zones is expected to endow joints with excellent seismic energy dissipation, large deformation capacity, and post-earthquake self-centering performance (e.g., crack closure and deformation recovery). The details of SMAF-ECC joint specimens are shown in Figure 1 [19,20].

Based on the above considerations, this study proposes a novel prefabricated self-centering frame joint incorporating SMAF-ECC. Compared with existing systems using SMA rods or hybrid SMA-steel reinforcement, the proposed SMAF-ECC joint offers distinct advantages [21,22]: (1) Cost-effectiveness: SMAF has lower production costs than continuous SMA rods and eliminates the need for expensive specialized anchoring devices. (2) Performance enhancement: The synergistic effect of SMAF and ECC improves not only self-centering and energy-dissipation capacities but also reduces residual deformation more effectively than single SMA or ECC reinforcement. (3) Constructability: SMAF can be directly mixed into ECC without additional cutting or complex installation procedures, simplifying the prefabrication process of joints.

To investigate the seismic performance and self-centering capacity of the novel joint, material mechanical property tests were first carried out. Building on the research group’s previous seismic performance tests on prefabricated concrete frame joints, numerical simulations on the seismic performance of the self-centering frame joint were conducted using ABAQUS 2022. Key performance indicators, including hysteretic curves, skeleton curves, stiffness degradation, residual displacement, and energy dissipation capacity, were analyzed, with particular attention paid to the influence of SMAF content on the joint’s self-centering capacity. This study performs a preliminary exploration on the application of SMAF-ECC composites in prefabricated structures, laying a foundation for subsequent experimental and theoretical research.

## 2. Prefabricated Concrete Self-Centering Frame Joint Design

### 2.1. Model Overview

**Figure 2:**
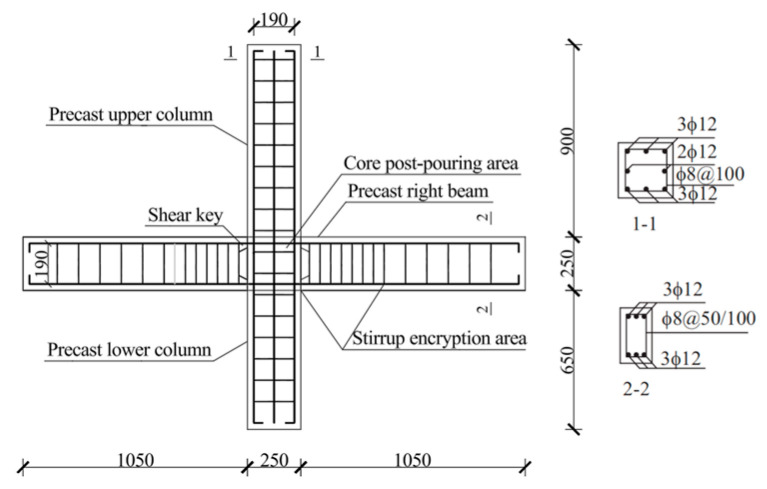
Joint dimensions and reinforcement diagram (unit: mm).

**Figure 3:**
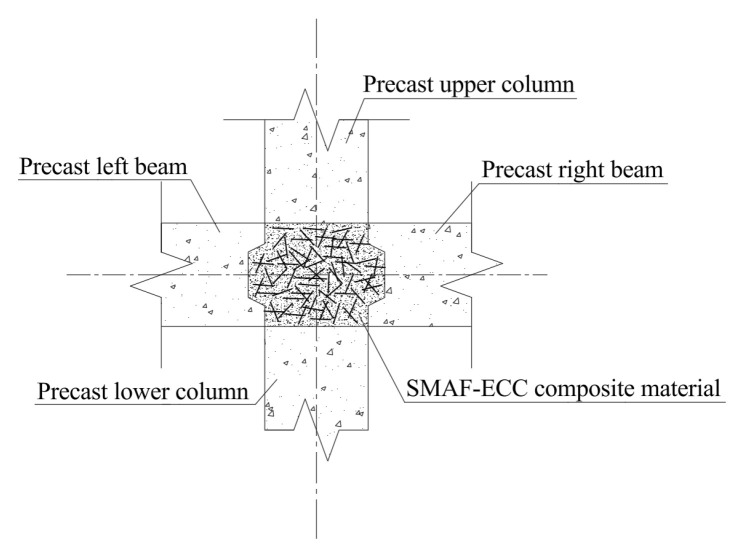
Self-centering frame joint structure diagram.

Based on the seismic performance tests of prefabricated concrete frame joints conducted by the research group [23], five prefabricated concrete frame joint models were established with different post-poured core area materials and SMAF volume content. The basic parameters of the joint models are listed in Table 1. The cross-sectional dimensions of the precast beam in the joint are 250 mm × 150 mm, and those of the precast column are 250 mm × 250 mm, both with a concrete cover thickness of 30 mm. The joint dimensions and strengthening detailing are shown in Figure 2, and the configuration schematic of the self-centering frame joint is presented in Figure 3.

### 2.2. Material Parameters

#### 2.2.1. SMAF

**Figure 4:**
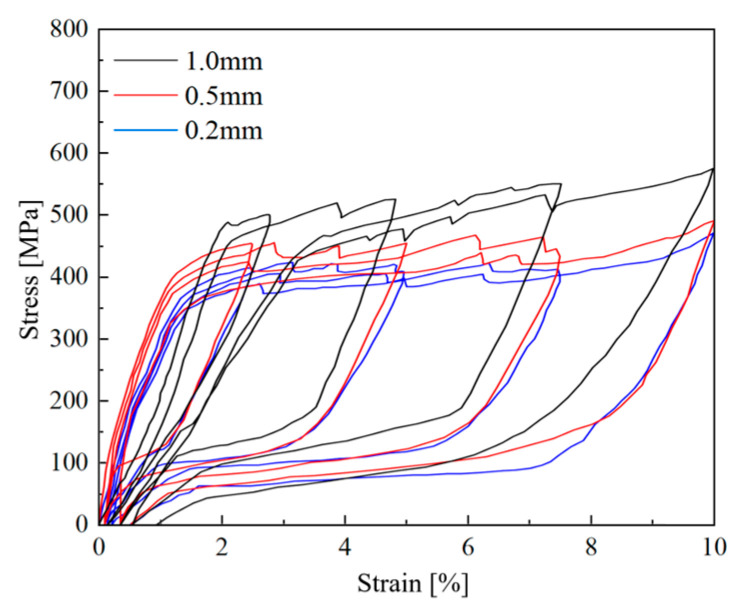
Stress–strain curves of SMAF.

The main compositions of SMAF are 55.86% Ni and 44.14% Ti. A WD-PD6305 universal testing machine (Jinan Liling Testing Machine Co., Ltd., Jinan, China) was used to conduct cyclic tensile tests on SMAF with diameters of 0.2 mm, 0.5 mm, and 1.0 mm, and a consistent length of 200 mm. Prior to testing, the SMAF were completely immersed in boiling water (100 °C) for 15 min, then removed and placed in cold water for another 15 min. A total of 15 such hot and cold cycles were performed to ensure the stability of the internal crystalline structure. The stress–strain curves obtained from the cyclic tensile tests are shown in Figure 4, and the cyclic tensile performance indices are presented in Table 2. As shown in Figure 4, the stress–strain curves of SMAF with different diameters all exhibit a large stress plateau and a distinct flag-shaped profile. After cyclic tension, the unloading residual strain of SMAF remains stable within 1%, indicating excellent self-recovery performance.

#### 2.2.2. ECC

ECC was prepared according to the mix proportion specified in Table 3, which was then poured into dog-bone-shaped thin-plate molds and standard cube molds, respectively, followed by vibratory compaction for molding [24]. The specimens were initially placed in a standard curing chamber for 24 h, followed by demolding and subsequent curing in a standard curing room for 28 days. After curing, uniaxial tensile tests were conducted on the dog-bone-shaped thin-plate specimens, while compressive strength tests were performed on the standard cube specimens. The measured mechanical properties of ECC are presented in Table 4. As shown in Table 4, ECC has an ultimate tensile strength of 4.27 MPa, an ultimate tensile strain of 6.13%, and a compressive strength of 35.7 MPa.

#### 2.2.3. Steel Bar and Concrete

The longitudinal reinforcement of the joints consists of HRB400 steel bars with a diameter of 12 mm, while the stirrups are HPB300 steel bars with a diameter of 6 mm. The mechanical properties of the steel bars obtained from tensile tests are presented in Table 5. Concrete was prepared in accordance with the mix proportion specified in Table 6, and standard cube compressive strength tests of the concrete were conducted. The measured compressive strength of the concrete is 32.9 MPa, meeting the C30 strength grade requirements.

## 3. Establishment and Verification of the Finite Element Model for Prefabricated Concrete Self-Centering Frame Joints

### 3.1. Constitutive Relationship of Materials

#### 3.1.1. Concrete

**Figure 5:**
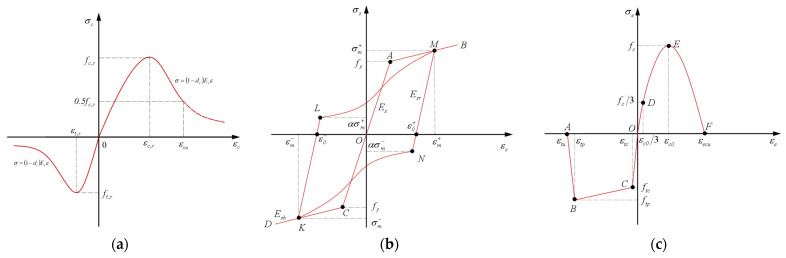
Material constitutive models: (**a**) Concrete [25,26]; (**b**) Steel bar [27]; (**c**) ECC [29].

Based on the failure mode of frame joints, the plastic damage model [25] is adopted. The stress–strain curve specified in Appendix C of GB 50010-2010 [26] is used to simulate the mechanical properties of concrete materials. Meanwhile, the damage factor and stiffness recovery coefficient are introduced to consider the elastic stiffness degradation and stiffness recovery effect of concrete under tension and compression. As shown in Figure 5a, the relevant formulas are expressed as follows:

The uniaxial tensile constitutive relationship:

(1) $$\sigma =\left(1-d_{t}\right)E_{c}\varepsilon$$

(2) $$d_{t}=\left\{\begin{array}{ccc} 1-\rho_{t}\left(1.2-0.2x^{5}\right) & x\leq 1 & \left(a\right)\\ 1-\frac{\rho_{t}}{\alpha_{t}{\left(x-1\right)}^{1.7}+x} & x>1 & \left(b\right) \end{array}\right.$$

where $x=\frac{\varepsilon }{\varepsilon_{t,r}}$; $\rho_{t}=\frac{f_{t,r}}{E_{c}\varepsilon_{t,r}}$; *E_c_* denotes the elastic modulus of concrete; *α_t_* represents the parameter of the descending section of the uniaxial tensile stress–strain curve of concrete; *f*_*t*,*r*_ stands for the uniaxial tensile strength of concrete; *ε*_*t*,*r*_ is the peak tensile strain of concrete corresponding to *f*_*t*,*r*_; *d_t_* refers to the tensile damage factor of concrete.

The uniaxial compression constitutive relationship:

(3) $$\sigma =\left(1-d_{c}\right)E_{c}\varepsilon$$

(4) $$d_{c}=\left\{\begin{array}{ll} 1-\frac{\rho_{c}n}{n-1+x^{n}} & x\leq 1\\ 1-\frac{\rho_{c}}{\alpha_{c}{\left(x-1\right)}^{2}+x} & x>1 \end{array}\right.$$

where $x=\frac{\varepsilon }{\varepsilon_{c,r}}$; $\rho_{t}=\frac{f_{c,r}}{E_{c}\varepsilon_{c,r}}$; $n=\frac{E_{c}\varepsilon_{c,r}}{E_{c}\varepsilon_{c,r}-f_{c,r}}$; $\alpha_{c}$ represents the parameter of the descending section of the uniaxial compressive stress–strain curve of concrete; $f_{c,r}$ denotes the representative value of the uniaxial compressive strength of concrete; $\varepsilon_{c,r}$ is the peak compressive strain corresponding to $f_{c,r}$; $d_{c}$ refers to the compressive damage factor of concrete.

#### 3.1.2. Steel Bar

Considering the bond-slip effect between concrete and steel bars, this study adopts the improved hysteretic model for reinforcement proposed by Fang et al. [27] to define the constitutive relationship of steel bars, as shown in Figure 5b. By adjusting the stiffness parameters of steel bars in the loading path, the model enables the steel bar to experience slight changes prior to significant variations during the bond-slip force transmission process, which is closer to the actual stress state of steel bars under service conditions and thus improves the accuracy of model analysis. In the figure: $\sigma_{m}^{+}$ and $\sigma_{m}^{-}$ represent the maximum tensile stress and maximum compressive stress, respectively; $\varepsilon_{m}^{+}$ and $\varepsilon_{m}^{-}$ denote the maximum tensile strain and maximum compressive strain, respectively; $\varepsilon_{0}^{+}$ and $\varepsilon_{0}^{-}$ stand for the strains at the intersections of the tensile and compressive unloading sections with the coordinate axis, respectively; α is the hysteretic energy dissipation influence coefficient; *E_s_* denotes the initial stiffness; *E_sh_* represents the hardening stiffness; and *E_sr_* refers to the unloading stiffness.

#### 3.1.3. SMAF

The superelastic constitutive model in ABAQUS software is adopted to simulate the mechanical behavior of superelastic SMAF. This constitutive model balances calculation efficiency and accuracy, with specific model parameters consistent with those reported in the literature [16,28].

#### 3.1.4. ECC

Consistent with the concrete simulation method, the plastic damage model is also adopted for ECC. The stress–strain relationship of ECC is based on the uniaxial compressive and tensile constitutive models of ECC established in [29]. Meanwhile, the damage factor and stiffness recovery coefficient are introduced to consider the elastic stiffness degradation and stiffness recovery effect of ECC under tension and compression. As shown in Figure 5c, the relevant formulas are expressed as follows:

The uniaxial tensile constitutive relationship:

(5) $$\sigma_{e}=\left\{\begin{array}{lll} E_{e}\varepsilon_{e} & 0\leq \varepsilon_{e}\leq \varepsilon_{tc} & \left(a\right)\\ f_{tc}+\left(f_{tp}-f_{tc}\right)\frac{\varepsilon_{e}-\varepsilon_{tc}}{\varepsilon_{tu}-\varepsilon_{tp}} & \varepsilon_{tc}\leq \varepsilon_{e}\leq \varepsilon_{tp} & \left(b\right)\\ f_{tp}\left(1-\frac{\varepsilon_{e}-\varepsilon_{tp}}{\varepsilon_{tu}-\varepsilon_{tp}}\right) & \varepsilon_{tp}\leq \varepsilon_{e}\leq \varepsilon_{tu} & \left(c\right) \end{array}\right.$$

where *E_e_* denotes the elastic modulus of ECC; $f_{tc}$ and $\varepsilon_{tc}$ represent the initial cracking stress and cracking strain of ECC under tension, respectively; $f_{tp}$ and $\varepsilon_{tp}$ stand for the peak stress and corresponding strain of ECC under tension, respectively; and $\varepsilon_{tu}$ refers to the ultimate strain of ECC under tension.

The expression of the tensile damage factor *d_t_* is as follows:

(6) $$d_{t}=\left\{\begin{array}{lll} 0 & 0\leq \varepsilon_{e}<\varepsilon_{tc} & \left(a\right)\\ 1-\sqrt{\frac{f_{tc}}{\varepsilon_{e}E_{e}}+\frac{\left(f_{tp}-f_{tc}\right)\left(\varepsilon_{e}-\varepsilon_{tc}\right)}{E_{e}\left(\varepsilon_{tp}-\varepsilon_{tc}\right)\varepsilon_{e}}} & \varepsilon_{tc}\leq \varepsilon_{e}<\varepsilon_{tp} & \left(b\right)\\ 1-\sqrt{\frac{f_{tp}\varepsilon_{tu}-f_{tp}\varepsilon_{e}}{E_{e}\left(\varepsilon_{tu}-\varepsilon_{tp}\right)\varepsilon_{e}}} & \varepsilon_{tp}\leq \varepsilon_{e}<\varepsilon_{tu} & \left(c\right) \end{array}\right.$$

The uniaxial compression constitutive relationship:

(7) $$\sigma_{e}=\left\{\begin{array}{lll} E_{e}\varepsilon_{e} & 0\leq \varepsilon_{e}<\frac{\varepsilon_{c0}}{3} & \left(a\right)\\ \frac{f_{c}}{3}\left[1+6\left(\frac{\varepsilon_{e}-\varepsilon_{c0}/3}{\varepsilon_{c0}}\right)\right]-4.5{\left(\frac{\varepsilon_{e}-\varepsilon_{c0}/3}{\varepsilon_{c0}}\right)}^{2} & \frac{\varepsilon_{c0}}{3}\leq \varepsilon_{e}<\varepsilon_{c0} & \left(b\right)\\ f_{c}\left[1-{\left(\frac{\varepsilon_{e}-\varepsilon_{ecu}/3}{\varepsilon_{ecu}-\varepsilon_{ecu}/3}\right)}^{2}\right] & \varepsilon_{c0}\leq \varepsilon_{e}<\varepsilon_{ecu} & \left(c\right) \end{array}\right.$$

where $f_{c}$ denotes the peak compressive strength of ECC; $\varepsilon_{c0}$ represents the strain corresponding to $f_{c}$; $\varepsilon_{ecu}$ refers to the ultimate compressive strain of ECC.

The expression of the compressive damage factor *d_c_* is as follows:

(8) $$d_{c}=\left\{\begin{array}{lll} 0 & 0\leq \varepsilon_{e}<\frac{\varepsilon_{c0}}{3} & \left(a\right)\\ 1-\sqrt{\frac{6f_{c}\varepsilon_{c0}\varepsilon_{e}-\varepsilon_{c0}^{2}f_{c}-3f_{c}\varepsilon_{e}}{2E_{e}\varepsilon_{c0}^{2}}} & \frac{\varepsilon_{c0}}{3}\leq \varepsilon_{e}<\varepsilon_{c0} & \left(b\right)\\ 1-\sqrt{\frac{3\left(\varepsilon_{c0}^{2}f_{c}-3f_{c}\varepsilon_{e}^{2}+2f_{c}\varepsilon_{c0}\varepsilon_{e}\right)}{4E_{c}\varepsilon_{e}\varepsilon_{c0}^{2}}} & \varepsilon_{c0}\leq \varepsilon_{e}<\varepsilon_{cu} & \left(c\right) \end{array}\right.$$

### 3.2. Mesh and Interaction

Concrete and ECC are modeled using the C3D8R element, while steel bars and SMAF are simulated with the T3D2 truss element [30]. Among them, the finite element formulation adopts the standard Lagrangian type; the shape function of the C3D8R element is a trilinear interpolation function, and the T3D2 element uses a linear shape function; the number of numerical integration points is 2 × 2 × 2 for full integration, and Jacobian points are calculated via Gaussian integration. Considering calculation efficiency, accuracy, and component dimensions, a structured meshing technique is employed. Following multiple trial adjustments and convergence analyses, the mesh dimensions for the frame joint are determined: the mesh size of steel bars and SMAF is 12.5 mm, and that of concrete and ECC is 25 mm. The contact model employs a hybrid approach combining the Coulomb friction model and cohesive zone model. By defining tangential and normal parameters, the mechanical behavior of the joint interface between precast concrete and cast-in-place ECC can be effectively simulated [31]. The reinforcement cage and SMAF are embedded in the overall model using the “embedded region” constraint.

### 3.3. Boundary Conditions and Load

**Figure 6:**
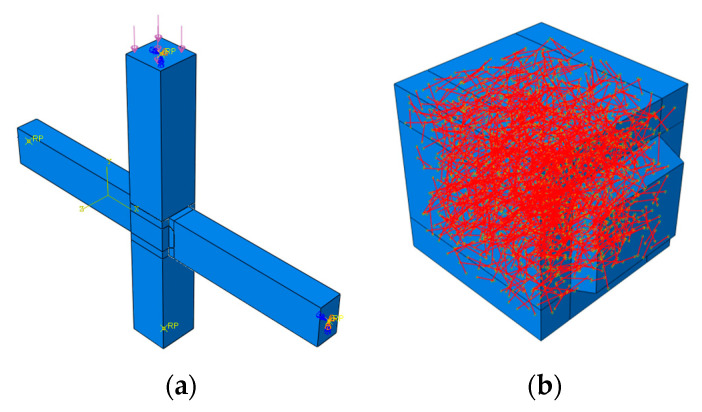
Finite element model. (**a**) Self-centering frame joint model; (**b**) Core area SMAF-ECC composite materia.

**Figure 7:**
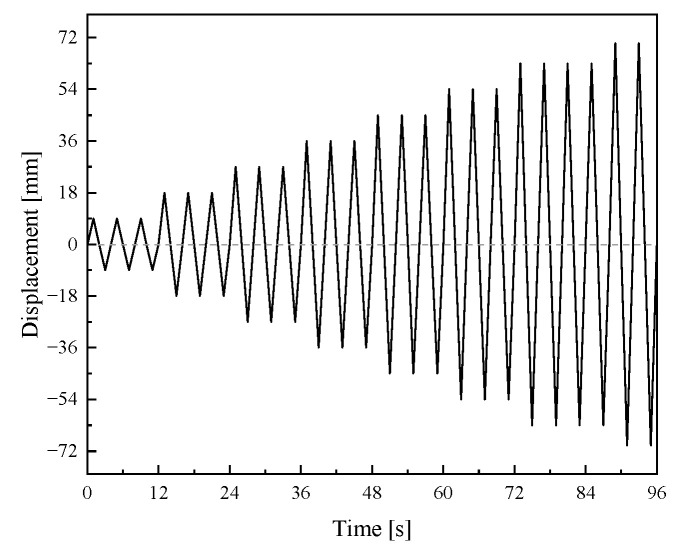
Loading protocol.

Referring to the loading scheme for the seismic performance test of prefabricated frame joints in the previous research of the research group [23], a vertical axial load of 750 kN is applied to the top of the column in the finite element model, maintaining a constant column axial compression ratio of 0.4. Cyclic reversed load is applied horizontally at the column top to simulate seismic action, as shown in Figure 6. Displacement-controlled loading is adopted in stages, including 8 loading stages with a displacement increment of 9 mm per stage, and each stage is cycled three times. The loading protocol is illustrated in Figure 7.

### 3.4. Model Verification

**Figure 8:**
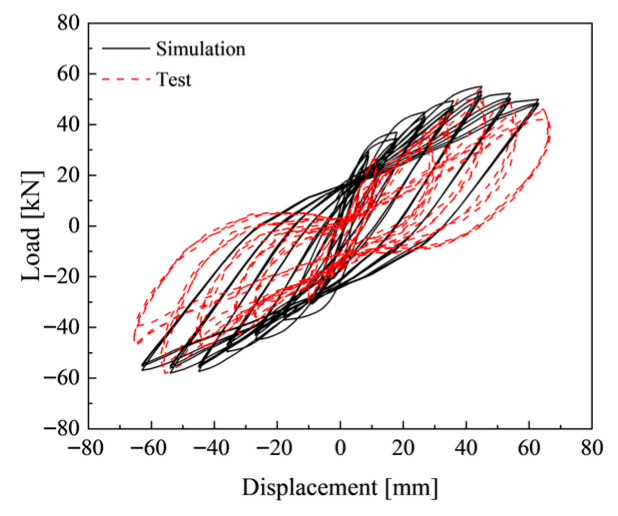
Hysteresis curve comparison diagram.

To address the concern regarding the reliability of conclusions derived from numerical simulation, experimental tests have been conducted to validate the proposed finite element model. Figure 8 is the comparison between the hysteretic curves obtained by finite element simulation and the test results of RC joints. It can be seen from Figure 6 that the RC joint test results are close to the simulation results. The peak load from finite element simulation is 55.03 kN, while that from the test is 52.4 kN. The error between the two is 4.78%. Furthermore, the trends of their hysteretic curves are consistent with each other. These results indicate that the simulation results are in good agreement with the test results. The adopted finite element model and material constitutive models in this study are reasonable and suitable for the seismic performance analysis of such self-centering frame joints.

## 4. Results Analysis

### 4.1. Hysteretic Curves

**Figure 9:**
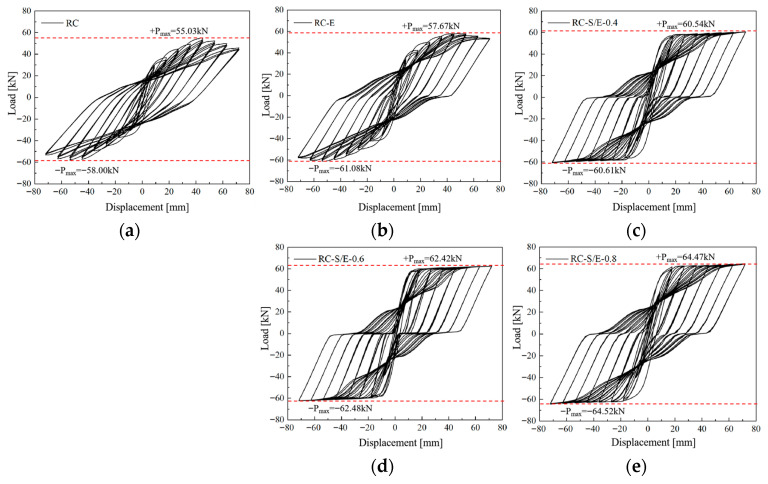
Load–displacement hysteresis curves of joint model. (**a**) RC; (**b**) RC-E; (**c**) RC-S/E-0.4; (**d**) RC-S/E-0.6; (**e**) RC-S/E-0.8.

The load–displacement hysteretic curves obtained from finite element simulations of the five joint models are presented in Figure 9. As illustrated in Figure 9, when the applied displacement is small, the envelope area of the hysteretic curves for each joint model is small. This is because under small column-end displacement loading conditions, the joints are in the elastic stage, and the hysteretic response is weak. As larger displacements are applied continuously, the envelope area of the hysteretic curves gradually increases, indicating that the five joints exhibit a certain energy dissipation capacity. Compared with RC and RC-E joints, the hysteretic curves of the three RC-S/E self-centering joints exhibit a distinct flag-shaped profile. Furthermore, their hysteretic loops are relatively plump, and the residual displacement of the joints after each cyclic unloading is small, demonstrating that the incorporation of SMAF improves the hysteretic performance of the joints. As the SMAF content increases from 0.4% to 0.8%, the hysteretic curves shift upward overall, and the restoring force gradually increases.

### 4.2. Skeleton Curves

**Figure 10:**
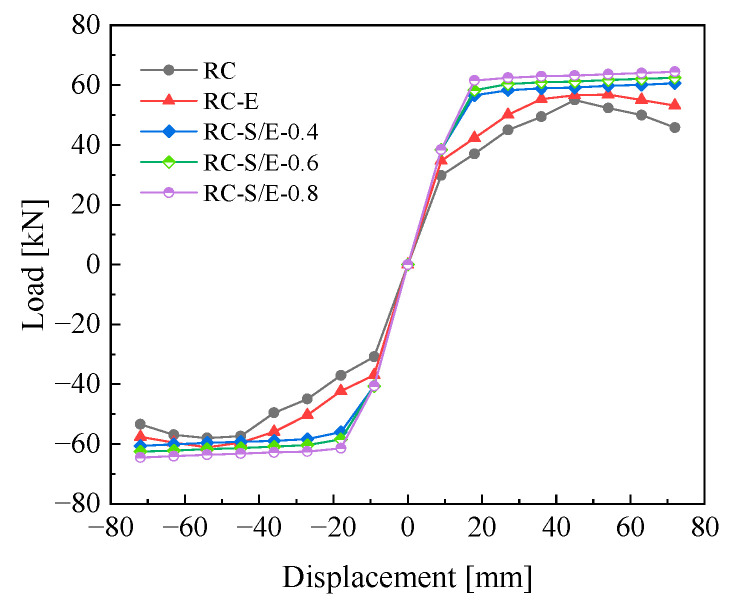
Skeleton curves.

The skeleton curves reflect the bearing capacity, stiffness, and ductility of the joint models at different loading stages. The skeleton curves obtained from finite element simulations of the five joint models are presented in Figure 10. In the initial loading stage, the five joint models exhibit high stiffness as they remain in the elastic stage. As the loading displacement increases, the slope of the skeleton curves of the joint models gradually decreases, their stiffness reduces, and the joints enter the plastic energy dissipation stage. The yield strength and ultimate bearing capacity of RC-E and RC-S/E joints are higher than those of RC joints. This is because the tensile and compressive strengths of ECC and SMAF-ECC composites are higher than those of C30 concrete. Replacing the core area concrete with these two composites enhances the shear capacity of the joint core area, thereby improving the flexural capacity of the joints. Notably, the bearing capacity of RC-S/E self-centering joints continues to increase slightly after reaching their yield strength, with an ultimate bearing capacity of 64.47 kN—17.15% and 11.78% higher than those of RC and RC-E joints, respectively. This demonstrates that incorporating SMAF into ECC not only further enhances the joints’ bearing capacity but also significantly improves their ductility, enabling the joints to sustain greater deformation prior to final failure. Comparing the skeleton curves of RC-S/E self-centering joints with different SMAF volume content indicates that increasing the SMAF volume content can enhance the joints’ bearing capacity.

### 4.3. Stiffness Degradation

**Figure 11:**
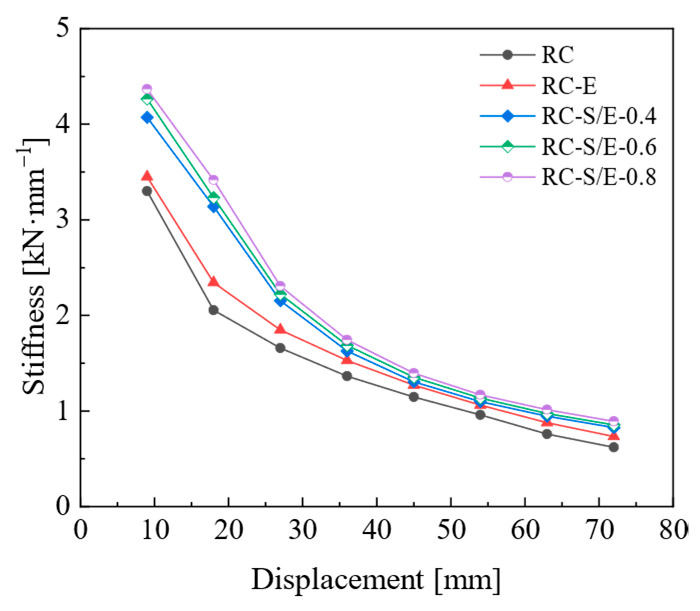
Stiffness degradation curves.

Structural stiffness degrades due to cumulative structural damage, and the stiffness of each joint is characterized by secant stiffness. The simulated stiffness degradation curves from finite element simulations are presented in Figure 11. As illustrated in Figure 11, the initial stiffness of the three RC-S/E self-centering joints is higher than that of RC and RC-E joints. In the early loading stage, the stiffness degradation rate of RC-S/E self-centering joints is relatively rapid; however, as the applied displacement increases, the degradation rate slows down for all joints. Throughout the loading process, the stiffness of RC-E and RC-S/E joints is higher than that of RC joints under the same applied displacement. This indicates that adopting ECC and SMAF-ECC composites helps enhance the stiffness of prefabricated frame joints. The stiffness degradation curves of RC-S/E self-centering joints with different SMAF volume content are nearly identical, indicating that increasing the SMAF volume content has a negligible effect on the joints’ stiffness degradation performance.

### 4.4. Residual Displacement

**Figure 12:**
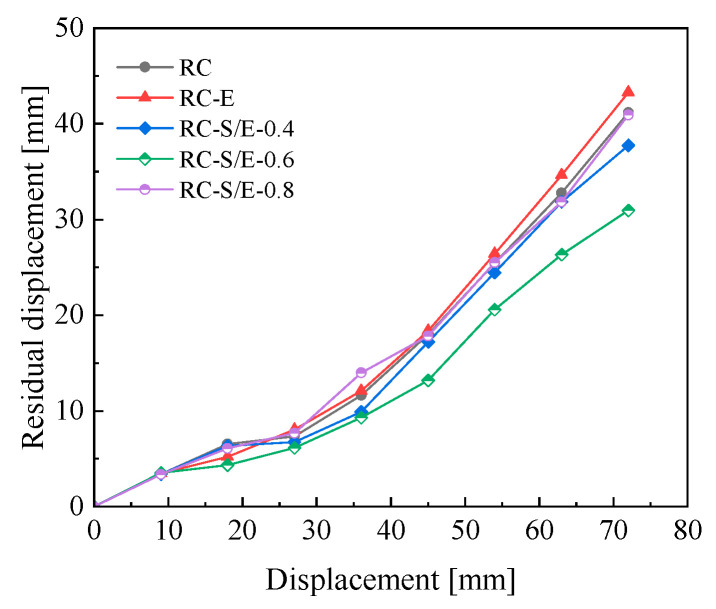
Residual displacement of column-end.

Residual displacements of the joints occur after each stage of cyclic unloading, and their magnitude directly reflects the self-centering performance of the joints. The column-end residual displacement curves of each joint from finite element simulations are presented in Figure 12. Under the same applied displacement, the column-end residual displacement of RC-S/E joints is significantly smaller than that of RC and RC-E joints, while that of RC-E joints is slightly larger than that of RC joints. When the column-end displacement reaches 72 mm, the recoverable deformation ratios of RC and RC-E joints are 43% and 40%, respectively, while that of RC-S/E-0.6 joints reaches 57% (corresponding to a column-end residual displacement of 30.96 mm). This indicates that adopting ECC in the core zone of prefabricated frame joints does not reduce the joints’ residual displacement. However, under large-displacement loading, the superelasticity of SMAF in the ECC matrix is effectively utilized, providing restoring force during joint unloading and significantly improving the joints’ self-centering performance. Comparing the residual displacement curves of RC-S/E self-centering joints with different SMAF volume content, it is found that the residual displacement of RC-S/E joints under all loading levels is the smallest when the SMAF volume content is 0.6%. This demonstrates that incorporating SMAF enhances the self-centering performance of prefabricated joints. However, an excessively high SMAF volume content leads to fiber agglomeration, which reduces the bonding strength between SMAF and the ECC matrix and consequently impairs the joints’ self-centering performance [32].

### 4.5. Energy Dissipation Capacity

**Figure 13:**
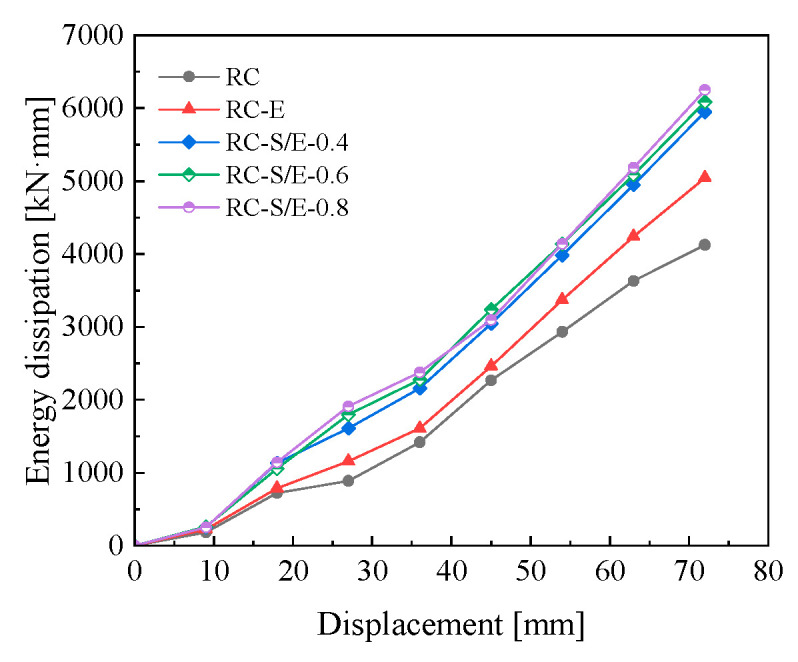
Energy dissipation curves.

Energy dissipation capacity is one of the important indicators for evaluating the seismic performance of structures or components. The area enclosed by the hysteretic curves directly reflects the energy dissipation capacity of structures. Figure 13 presents the energy dissipation curves of the five joint models from finite element simulations. As illustrated in Figure 13, at the initial loading stage, the energy dissipation capacity of the joint is low, and their energy dissipation curves almost overlap. This is because the applied displacement is small, and the differences in the energy dissipation performance of different materials are not fully exhibited. As the applied displacement gradually increases, the energy dissipation capacity of each joint also gradually increases, and significant differences arise among the energy dissipation capacities of different joints. Among them, the slopes of the energy dissipation curves of the three RC-S/E self-centering joints are relatively large, while that of the RC joints is the smallest. As the SMAF volume content increases, the energy dissipation capacity of the RC-S/E self-centering joints is further enhanced. When loaded to 72 mm, the energy dissipation of RC-S/E-0.8 joints is 51.29% and 23.79% higher than that of RC and RC-E joints, respectively. This demonstrates that SMAF in the ECC matrix significantly enhances the energy dissipation capacity of prefabricated frame joints by utilizing its flag-shaped hysteretic behavior under cyclic loading.

## 5. Conclusions

This study proposes a self-centering prefabricated frame joint based on SMAF-ECC composites. Based on material mechanical property tests, ABAQUS was employed to simulate the seismic performance of the self-centering joints, and the influence of SMAF volume content on the joint seismic performance was systematically analyzed.

(1) Replacing the concrete in the core zone of prefabricated concrete frame joints with SMAF-ECC composites can significantly reduce the residual displacement of the joints after unloading, with a maximum recoverable deformation ratio of 57%, thus endowing the joints with excellent self-centering performance.
(2) SMAF-ECC composites can optimize the hysteretic performance of the joints and enhance their bearing capacity, stiffness, and energy dissipation capacity. Compared with traditional reinforced concrete (RC) prefabricated concrete frame joints, the ultimate bearing capacity of SMAF-ECC-reinforced self-centering prefabricated concrete frame joints can be increased by up to 17.15%, and the cyclic energy dissipation capacity under single-stage loading can be increased by a maximum of 51.29%.
(3) Increasing SMAF volume content can effectively improve the bearing capacity and energy dissipation capacity of the joints, but exerts a negligible influence on the secant stiffness. Specifically, when the SMAF volume content does not exceed 0.6%, it facilitates reducing the residual displacement of self-centering joints. When the volume content exceeds 0.6%, the excessive content may lead to the deterioration of the self-centering performance.

However, this study still has certain limitations: First, the research results are based on numerical simulations, lacking comprehensive experimental validation (e.g., quasi-static tests on full-scale specimens) to verify the reliability of the simulation results; second, the influence of local SMAF concentration (which may occur during mixing and pouring) on joint performance was not considered in the simulation.

In terms of engineering applications, the SMAF-ECC self-centering prefabricated joint proposed in this study provides a new technical solution for improving the seismic resilience of prefabricated structures. It is particularly suitable for prefabricated buildings in high-seismic-intensity areas, which can help reduce post-earthquake repair costs and shorten functional recovery time, contributing to the promotion of the industrialized construction of resilient structures.

Directions for further research are as follows: (1) Conduct full-scale specimen tests to verify the seismic performance and self-centering effect of SMAF-ECC joints, and modify and optimize the numerical simulation model based on test results. (2) Explore the influence of key factors such as SMAF distribution uniformity, different loading systems (e.g., dynamic cyclic loading) on joint performance. (3) Optimize the mix ratio of SMAF-ECC and the structural details of the joint to further improve its engineering applicability and economy.

## Figures and Tables

**Table 1:** Basic parameters of joint model.

Model Number	Longitudinal Reinforcement Model	Stirrup Model	Post-Poured Core Area Material	SMAF Diameter [mm]	SMAF Length [mm]	SMAF Volume Content [%]
RC	HRB400	HPB300	C30 concrete	—	—	—
RC-E			ECC	—	—	—
RC-S/E-0.4			SMAF-ECC	0.5	50	0.4
RC-S/E-0.6			SMAF-ECC	0.5	50	0.6
RC-S/E-0.8			SMAF-ECC	0.5	50	0.8

**Table 2:** Mechanical properties of SMAF.

SMAF Diameter [mm]	Loading Strain [%]	Residual Strain [%]
0.2	2.5	0.10
	5	0.14
	7.5	0.22
	10	0.47
0.5	2.5	0.17
	5	0.23
	7.5	0.40
	10	0.71
1.0	2.5	0.22
	5	0.30
	7.5	0.55
	10	0.93

**Table 3:** ECC quality mix proportion.

Materials	Cement [kg]	Fly Ash [kg]	Quartz Sand [kg]	Water [kg]	PVA * [%]	Water Reducing Agent [kg]
Proportion	1.00	4.00	1.00	1.00	2	0.0079

* PVA fibers content is the volume content.

**Table 4:** ECC mechanical properties index.

Initial Cracking Stress [MPa]	Initial Crack Strain [%]	Ultimate Tensile Strength [MPa]	Limit Tensile Strain [%]	Compressive Strength [MPa]
2.57	0.36	4.27	6.13	35.7

**Table 5:** Mechanical properties of steel bar.

Category	Yield Strength [MPa]	Ultimate Strength [MPa]	Elastic Modulus [GPa]	Elongation [%]	Yield Strain [*με*]
HPB300	325	413	2.00 × 10^2^	21.6	1200
HRB400	369	457	2.01 × 10^2^	22.3	1920

**Table 6:** Concrete quality mix proportion (unit: kg).

Materials	Cement	Water	River Sand	Gravel
Proportion	1.00	0.55	1.83	3.40

## Data Availability

The original contributions presented in this study are included in the article. Further inquiries can be directed to the corresponding author.
